# Contribution of the μ‐opioid receptor system to affective disorders in temporal lobe epilepsy: A bidirectional relationship?

**DOI:** 10.1111/epi.17463

**Published:** 2022-12-28

**Authors:** Daichi Sone, Marian Galovic, Jim Myers, Georg Leonhardt, Ilan Rabiner, John S. Duncan, Matthias J. Koepp, Jacqueline Foong

**Affiliations:** ^1^ Department of Clinical and Experimental Epilepsy University College London Queen Square Institute of Neurology London UK; ^2^ Department of Psychiatry Jikei University School of Medicine Tokyo Japan; ^3^ Department of Neurology, Clinical Neuroscience Center University Hospital Zurich Zurich Switzerland; ^4^ Faculty of Medicine Imperial College London London UK; ^5^ Department of Neurosurgery Carl Gustav Carus University Hospitals Dresden Germany; ^6^ Invicro London UK

**Keywords:** affective disorders, endogenous opioid, opioid receptor, positron emission tomography, temporal lobe epilepsy

## Abstract

**Objective:**

Affective disorders are frequent comorbidities of temporal lobe epilepsy (TLE). The endogenous opioid system has been implicated in both epilepsy and affective disorders, and may play a significant role in their bidirectional relationship. In this cross‐sectional study, we investigated the association between μ‐opioid receptor binding and affective disorders in patients with TLE.

**Methods:**

Nine patients with TLE and depression/anxiety underwent ^11^C‐carfentanil positron emission tomography (CFN PET) and neuropsychiatric assessment, including the Hospital Anxiety and Depression Scale and the Positive and Negative Affect Schedule. The normalized CFN PET scans were compared with those of 26 age‐matched healthy controls. Correlation analyses with affective symptoms were performed by region of interest‐based analysis focusing on the limbic circuit and orbitofrontal cortex.

**Results:**

We observed widely reduced CFN binding potential (BP) in bilateral frontal lobes and striata in patients with TLE compared to healthy controls. In the TLE group, more severe anxiety and negative affect were associated with decreased CFN BP in the posterior cingulate gyrus.

**Significance:**

In patients with TLE, interictally reduced binding in the opioid system was associated with higher levels of anxiety and negative affect. We speculate that seizure‐related agonist‐driven desensitization and downregulation of opioid receptors could be a potential underlying pathomechanism.

**Key Points**

- The endogenous opioid neurotransmitter system has an important role in both epilepsy and affective disorders.
- Studies also suggested ictal opioid release and subsequent hyperexpression of opioid receptors.
- Using CFN PET, we found widely reduced CFN binding potentials in patients with TLE and affective symptoms.
- This study suggested the potential involvement of opioid systems in affective disorders in TLE.

## 1 INTRODUCTION

Affective disorders, in particular depression and anxiety,^1^, ^2^ are frequent comorbidities in patients with epilepsy. These affect approximately 20%–30% of patients with temporal lobe epilepsy (TLE)^3^, ^4^ and critically impact their quality of life.^5^ Individuals with depression have an increased risk of developing epilepsy, and may have a poor response to pharmacotherapy for seizures.^6^ The underlying biological mechanisms of this bidirectional association are unknown.

The opioid neurotransmitter system has an important role in emotion regulation and stress response. ^11^C‐Carfentanil (CFN), a selective μ‐opioid receptor subtype 1 agonist,^7^ and ^11^C‐diprenorphine, a nonselective opioid receptor antagonist, are positron‐emitting radiopharmaceuticals, which reversibly bind to either μ‐opioid receptors selectively (CFN) or reflect alterations at all the opioid receptors except opioid receptor‐like receptor, that is, μ, κ, and/or δ.^8^ Differences in binding potential of these positron emission tomography (PET) tracers reflect changes in opioid receptor density, or occupancy of the receptor by endogenous transmitters (endorphins, enkephalins, and endomorphins).

The endogenous opioid system has been implicated in both affective disorders and epilepsy.^6^ Decreased opioid receptor binding with positive mood after enjoyable stimuli such as a movie or music, corresponding to an endogenous opioid release, was demonstrated in the orbitofrontal and mesiotemporal areas including the amygdala.^9^ Conversely, increased μ‐opioid receptor binding, suggestive of reduced endogenous opioid release, was seen in the anterior cingulate, ventral pallidum, amygdala, and inferior temporal cortex during a sustained negative emotion state.^10^ Further evidence points to a dysregulation of the opioid neurotransmitter system during emotion regulation in patients with depression. Paradoxically, patients with depression presented with decreased μ‐opioid receptor binding, suggestive of either a downregulation of μ‐opioid receptors or increased endogenous opioid release in response to a sustained negative emotion state.^11^ This might represent a pathological stress response to negative emotion stimuli in depression. A recent retrospective PET study in healthy controls showed reduced CFN binding with subclinical depression and anxiety scores in cortical and subcortical areas, notably in amygdala, hippocampus, ventral striatum, and orbitofrontal and cingulate cortices.^12^

In patients with epilepsy, PET studies implicated a role of the opioid system in the termination of seizures, suggesting an ictal release of opioidlike substances.^13^ This ictal opioid release leads to a subsequent hyperexpression of opioid receptors.^13^ These adaptive processes start within hours after seizures and are likely to persist for several days. At a later stage, lateralized increases of opioid receptor binding were observed in the ipsilateral temporal lobe in patients with TLE, pointing to an interictally altered opioid system.^14^, ^15^ The explanation for relatively increased binding in the temporal neocortex and reduced binding in the amygdala in TLE remains unclear.^15^ Postictal and interictal changes of opioid transmission might lead to affective dysregulation, as reflected in impaired emotional processing in TLE patients.^16^, ^17^

Given these shared disturbances in affective disorders and epilepsy, we hypothesized a role of the opioid neurotransmitter system in the pathophysiology of interictal depression. We investigated the relationships between μ‐opioid receptor binding and affective disorders in patients with TLE using CFN PET. We aimed to detect alterations of the opioid system affecting the limbic circuit and orbitofrontal cortex, that is, areas that are crucial for emotion regulation.^9^, ^10^ Based on our observations and previous data, we propose a hypothetical model for the pathophysiology of interictal depression.

## 2 MATERIALS AND METHODS

### 2.1 Participants

We recruited nine consecutive patients with TLE (mean ± SD age = 42.7 ± 10.1 years, four females) from the neurology and neuropsychiatry clinics at the National Hospital for Neurology and Neurosurgery London, and the details are shown in Table 1. The lateralization and localization of epilepsy were determined by qualified neurologists based on seizure semiology and ictal/interictal electroencephalographic recordings. We excluded cases with (1) pregnancy or breastfeeding, (2) current or recent (<6 months) treatment with opioids, (3) alcohol consumption of >21 units per week, or (4) a history of drug abuse or use of illegal drugs. All patients were assessed by a consultant neuropsychiatrist (J.F.) via a clinical interview. Nine patients diagnosed with current or past mood and anxiety disorders, fulfilling criteria according to the Diagnostic and Statistical Manual of Mental Disorders, 4th edition revised, were recruited into the study. At the time of the PET scan, depressive and anxiety symptoms were evaluated using the Hospital Anxiety and Depression Scale (HADS). Additionally, the Positive and Negative Affect Scale (PANAS) was used to measure the two affective dimensions.^18^ All patients underwent 3‐T magnetic resonance imaging (MRI) scans, which identified no structural lesions except for one case with unilateral hippocampal sclerosis (Patient 7). We obtained written informed consent from all patients, and the study protocol was approved by the London‐Bromley Research Ethics Committee (15/LO/1767).

**TABLE 1:** Clinical demographics of patients with temporal lobe epilepsy in this study

Patient #	Focus side	Age, years	Sex	Onset age, years	Sz types	Sz/month	Psychiatric episode	HADS‐D	HADS‐A	PANAS positive	PANAS negative	ASMs	Psychoactive drugs	Interval from last Sz, days
1	L	34	F	13	FAS, FBTCS	.1	Depression, anxiety	3	13	32	11	OXC	Sertraline	161
2	R	37	M	3	FAS, FIAS, FBTCS	114	Depression	16	13	12	12	CBZ, LEV, CLB	None	3
3	L	46	M	27	FAS, FIAS, FBTCS	43	Depression, anxiety	11	13	20	17	LEV, ZNS	Sertraline	1
4	L	56	F	37	FAS, FIAS, FBTCS	5	Episodes of low mood, anxiety	6	16	33	15	PGB, LTG, CLB, PER	None	1
5	L	36	M	34	FAS, FIAS	4	Depression, low mood	7	11	25	12	CBZ	None	26
6	L	27	M	13	FIAS, FBTCS	6	Depression, OCD in remission	10	13	28	16	LTG, PER, LCM, CLB	Mirtazapine	14
7	L	50	M	1	FIAS, FBTCS	100	Postictal depression	4	5	32	10	LCM, LTG, LEV	None	.1
8	R	56	F	15	FAS, FIAS	8	Postictal depression	1	5	25	12	OXC, LCM, PER	None	1
9	R	42	F	27	FIAS, FBTCS	2	History of anxiety	3	7	34	8	LTG, ZNS, PGB	None	2

Abbreviations: ASM, antiseizure medication; CBZ, carbamazepine; CLB, clobazam; F, female; FAS, focal aware seizures; FBTCS, focal to bilateral tonic–clonic seizures; FIAS, focal impaired awareness seizures; HADS, Hospital Anxiety and Depression Scale; HADS‐A, HADS‐Anxiety; HADS‐D, HADS‐Depression; LCM, lacosamide; LEV, levetiracetam; LTG, lamotrigine; M, male; OCD, obsessive–compulsive disorder; OXC, oxcarbazepine; PANAS, Positive and Negative Affect Scale; PER, perampanel; PGB, pregabalin; Sz, seizure(s); ZNS, zonisamide.

As a comparison group, we used data from 26 age‐matched healthy controls (mean ± SD age = 39.9 ± 10.0 years, all males) that were previously acquired on the same scanner in other studies.^19^, ^20^

### 2.2 Image acquisition

All PET/computed tomography (CT) scans were performed on a Siemens Healthcare HiRez 6 scanner. A low‐dose CT scan was performed for attenuation correction. CFN was injected intravenously (mean ± SD dose = 211 ± 74 MBq, range = 110–330 MBq), followed by the PET/CT scan; the emission scan duration was 90 min in 26 frames (8 × 15 s, 3 × 60 s, 5 × 120 s, 5 × 300 s, and 5 × 600 s, to a total of 5400 s). Three‐dimensional volumetric T1‐weighted structural MRI was also obtained in each participant by the following protocol: a magnetization prepared rapid gradient echo sequence (repetition time = 2300 ms, echo time = 2.98 ms, inversion time = 900 ms, flip angle = 9°, field of view = 256 mm, image matrix = 240 × 256) with an isotropic resolution of 1 mm (Magnetom Trio Syngo MR B13, Siemens, 3 T).

### 2.3 Image processing

**FIGURE 1:**
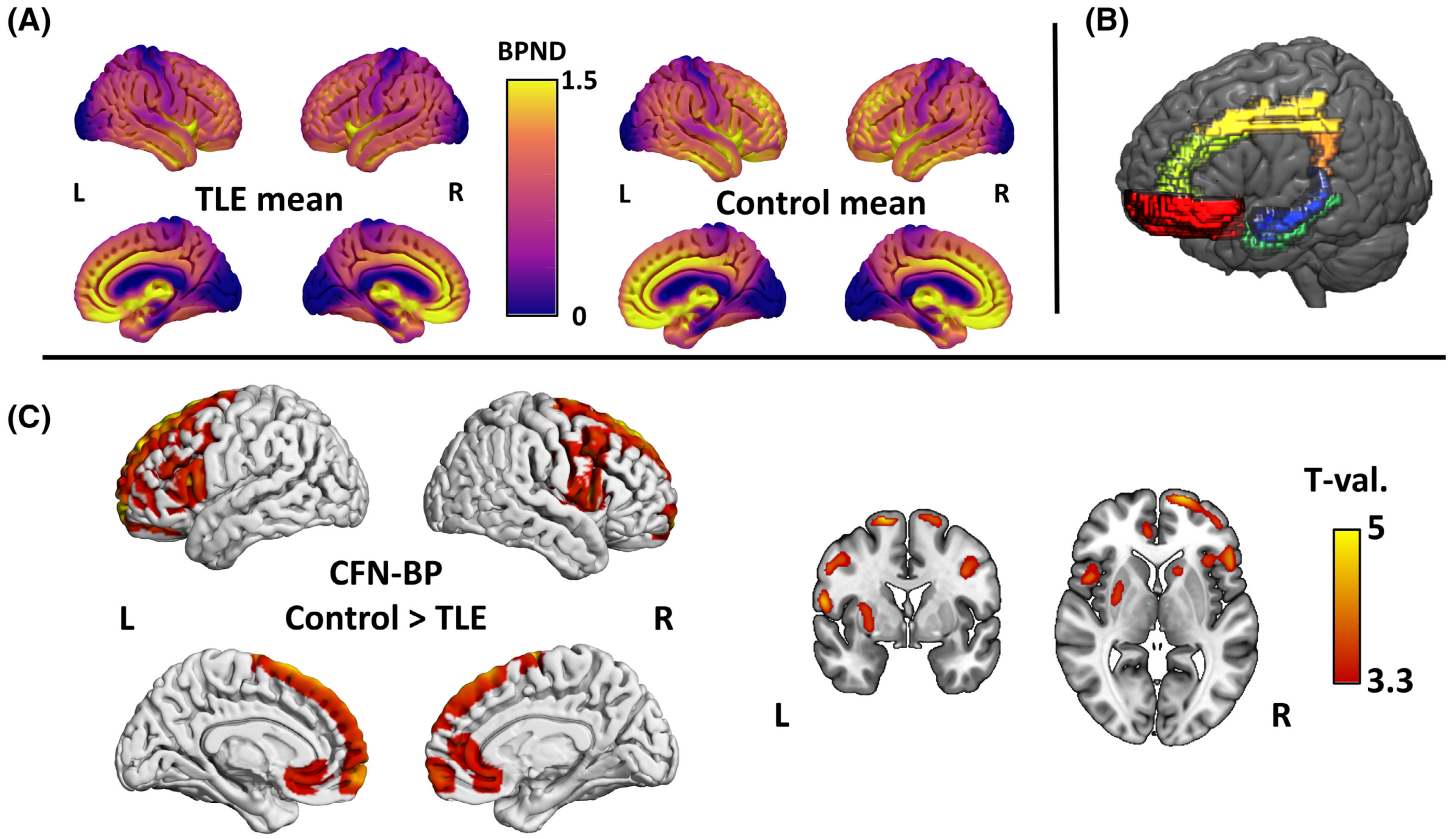
(A) The mean nondisplaceable binding potential (BPND) images of ^11^C‐carfentanil (CFN) positron emission tomography within each the temporal lobe epilepsy (TLE) and healthy control groups. (B) The location of volumes of interests in this study focusing on limbic circuit and orbitofrontal cortex. Red, orbitofrontal cortex; light green, anterior cingulate gyrus; yellow, middle cingulate gyrus; orange, posterior cingulate gyrus; dark blue, amygdala; blue, hippocampus; green, parahippocampal gyrus. Details are described in the Materials and Methods section. (C) Whole‐brain voxelwise comparison with age as a covariate revealed widespread reduction of CFN BPND within bilateral frontal lobes and striatum in TLE with height uncorrected *p* < .001 (*T* = 3.37) and cluster *p* < .05 with familywise error correction. L, left; R, right.

The dynamic PET data of patients with TLE and healthy controls were processed by MIAKAT software (www.miakat.org) and Statistical Parametric Mapping 12 (SPM12; http://www.fil.ion.ucl.ac.uk/spm/). Initially, the PET images underwent attenuation correction and motion correction by frame‐by‐frame realignment. For motion correction, large movements detected on video resulted in frame realignments or exclusion of frames. We also confirmed that the global motion (between the first and last frames) was <5 mm. After coregistration to the structural MRI, the nonlinear deformation parameters derived from unified segmentation of MRI were applied to the PET images. The nondisplaceable binding potential (BPND) values of CFN were quantified by the simplified reference tissue model with occipital lobe gray matter as the reference.^21^, ^22^ The mean images of BPND values in TLE and healthy control groups are shown in Figure 1A.

### 2.4 Voxel‐based analysis

We calculated whole‐brain voxelwise differences between TLE patients and healthy controls in CFN BPND using spatially normalized CFN BPND images smoothed with an 8‐mm full width at half maximum Gaussian kernel. We compared groups using a two‐sample *t*‐test design in SPM12, with age as a covariate.

### 2.5 Region of interest analysis

Based on previous literature,^9^, ^10^ we expected to detect changes in the limbic circuit and orbitofrontal areas. Thus, we used the following region of interests (ROIs) in this study: amygdala, hippocampus, parahippocampal gyrus, anterior cingulate gyrus, middle cingulate gyrus, posterior cingulate gyrus, and orbitofrontal cortex (OFC). These ROIs were extracted for the left and right hemisphere from the Automated Anatomical Labeling atlas,^23^ and the locations of ROIs are shown in Figure 1B. The mean BPND values within the gray matter of each ROI were calculated by the PETPVE12 toolbox,^24^ in the unsmoothed, spatially normalized CFN BPND images.

To determine seizure activity‐related alterations of the opioid system, we analyzed values ipsi‐ and contralateral to the focus in TLE separately.

### 2.6 Statistics

We compared ipsilateral, contralateral, and control BPND values using one‐way analysis of variance and the post hoc Tukey test. In addition, we assessed the correlation between BPND in each ROI and the scores of HADS and PANAS using Pearson correlation coefficient in the TLE group. We also assessed correlations between BPND with gender, age, age at seizure onset, duration of epilepsy, presence of focal aware seizures or focal to bilateral tonic–clonic seizures, log10 of interval since last seizure in days, and log10 of overall seizure frequency. The Shapiro–Wilk test did not detect significant deviations from a normal distribution for these variables. A *p*‐value < .05 was deemed significant. For voxelwise comparisons with SPM, we used an uncorrected threshold of *p* < .001 at the voxel level combined with a familywise error‐corrected *p* < .05 at the cluster level. SPSS software, version 25.0, was used for statistical analyses.

## 3 RESULTS

### 3.1 Demographics and psychiatric symptoms in patients with TLE

Clinical data including psychiatric episodes and affective scales are shown in Table 1. All the patients had a history of psychiatric comorbidities (Table 1). Three showed HADS‐Depression (HADS‐D) scores > 8 points, a frequently used cutoff for depression. Six patients showed raised anxiety scores (HADS‐Anxiety [HADS‐A] > 8 points).

### 3.2 Voxel‐based and ROI‐based comparison of CFN binding between TLE and healthy controls

**FIGURE 2:**
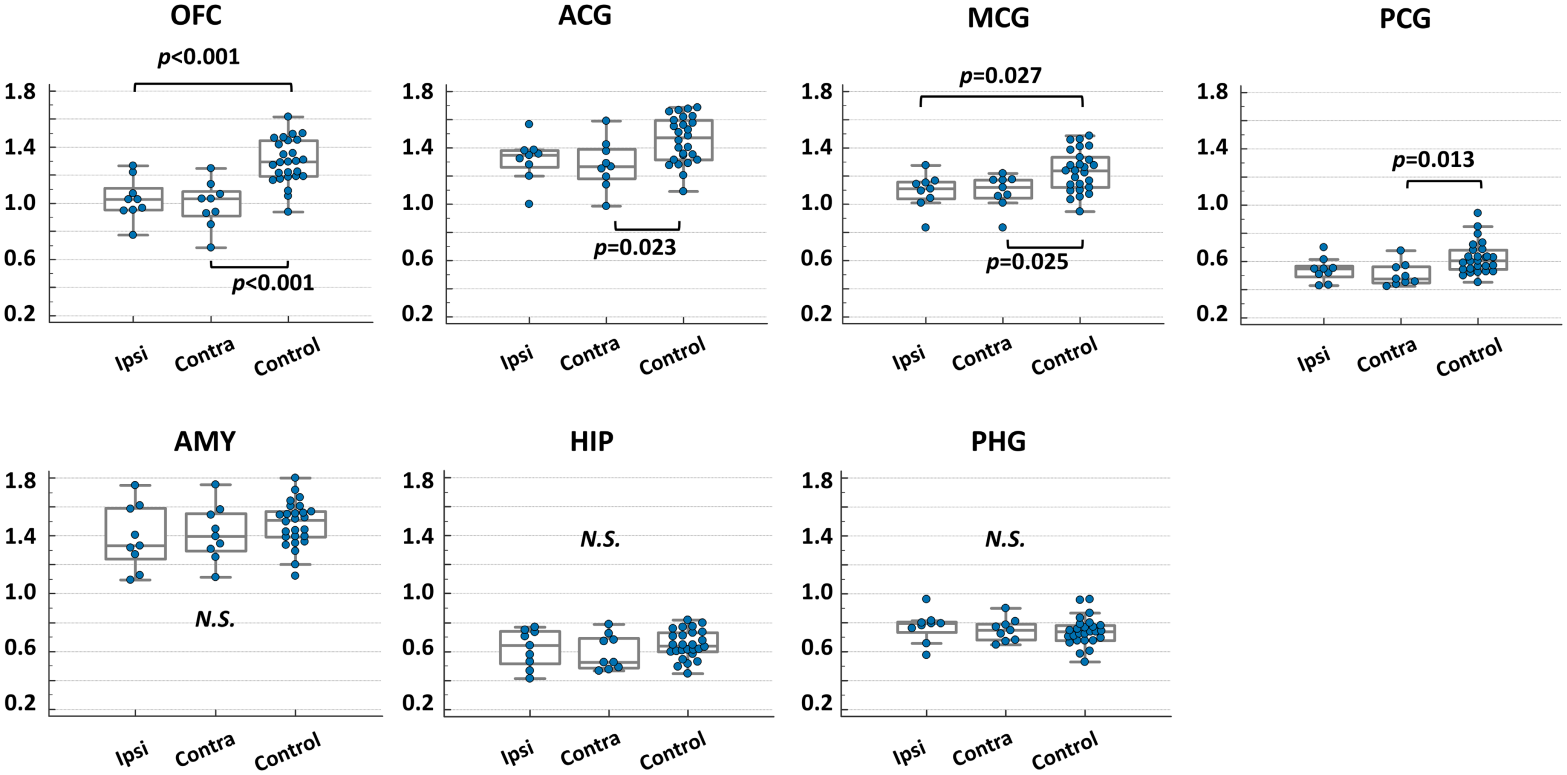
Group comparison of nondisplaceable binding potential of ^11^C‐carfentanil positron emission tomography in each volume of interest, among ipsilateral, contralateral, and control values. ACG, anterior cingulate gyrus; AMY, amygdala; HIP, hippocampus; MCG, middle cingulate gyrus; N.S., not significant; OFC, orbitofrontal cortex; PCG, posterior cingulate gyrus; PHG, parahippocampal gyrus.

The voxelwise group comparison revealed a widespread reduction of CFN BPND mainly in the frontal lobes and basal ganglia, including caudate and putamen (Figure 1C and Table 2) in patients with TLE, all of whom had current or past comorbid affective symptoms. There were no areas of increased CFN BPND in the TLE group. These findings were confirmed using ROI analysis in the OFC and cingulate gyri (Figure 2). The reductions were bilateral and did not differ between the ipsi‐ and contralateral hemisphere in patients with TLE. There was no difference in CFN BPND within the mesial temporal structures between patients with TLE and healthy controls. The effect sizes and percentages of reductions for each ROI are shown in Table 3. Furthermore, each patient's CFN BPND is presented in Figure S1.

**TABLE 2:** Detailed voxelwise results of reduced CFN BP in TLE

Regions of peaks	Cluster size	Cluster *p* (FWE)	*x*	*y*	*z*	*T*	Height *p* (FWE)
Left anterior prefrontal cortex (BA10), orbitofrontal cortex (BA11), premotor cortex/supplementary motor area (BA6), dorsolateral prefrontal cortex (BA9), Broca (Pars Triangularis) (BA45), frontal eye fields (BA8) Right premotor cortex/supplementary motor area (BA6), frontal eye fields (BA8), anterior prefrontal cortex (BA10)	13 037	<.001	−15	63	−9	6.82	.001
			−12	36	52	6.14	.003
			10	27	60	5.84	.006
			−16	16	64	5.78	.007
			16	−3	70	5.64	.011
			16	36	54	5.63	.011
			10	45	48	5.58	.012
			−9	10	69	5.45	.016
			−10	22	63	5.27	.025
			−18	50	38	5.23	.028
			−10	54	38	5.13	.035
			−51	20	10	5.01	.047
			10	64	−10	4.97	.052
			−10	62	22	4.93	.057
			27	24	52	4.71	.093
			−28	26	50	4.68	.099
Right Broca (Pars Opercularis) (BA44), primary motor cortex (BA4), frontal eye fields (BA8), premotor cortex/supplementary motor area (BA6), putamen	3969	.002	54	10	15	5.13	.036
			46	12	8	4.96	.053
			58	−2	14	4.62	.113
			42	6	40	4.48	.154
			44	16	46	4.45	.163
			50	12	32	4.13	.307
			54	−4	38	4.09	.334
			28	−6	−3	3.86	.485
			30	3	9	3.79	.542
Left orbitofrontal cortex (BA11), caudate, dorsal anterior cingulate cortex (BA32) Right anterior prefrontal cortex (BA10), orbitofrontal cortex (BA11)	1899	.025	−21	42	−15	4.79	.079
			9	46	9	4.22	.262
			−8	21	−9	4.11	.323
			−10	16	−3	4.02	.377
			3	28	−12	3.84	.500
			−4	39	−3	3.42	.808

*Note*: Coordinates are shown in Montreal Neurological Institute space. The height threshold for uncorrected *p* < .001 was *T* = 3.37. All local maxima at least 8 mm apart are shown.

Abbreviations: BA, Brodmann area; BP, binding potential; CFN, ^11^C‐carfentanil; FWE, familywise error; TLE, temporal lobe epilepsy.

**TABLE 3:** Mean/SD values and effect sizes of ROI‐based CFN BP in the group comparisons

Region	Ipsilateral				Contralateral				HC	
	Mean	SD	Reduction, %	Cohen *d*	Mean	SD	Reduction, %	Cohen *d*	Mean	SD
OFC	**1.03**	**.15**	**21%**	**−1.74**	**.99**	**.17**	**24%**	**−1.88**	1.30	.16
ACG	1.31	.15	10%	−.90	**1.28**	**.17**	**12%**	**−1.03**	1.45	.16
MCG	**1.09**	**.12**	**12%**	**−1.10**	**1.09**	**.12**	**12%**	**−1.10**	1.24	.15
PCG	.54	.08	13%	−.83	**.51**	**.08**	**19%**	**−1.14**	.62	.11
AMY	1.39	.22	6%	−.48	1.42	.19	4%	−.35	1.48	.15
HIP	.62	.13	4%	−.26	.59	.12	8%	−.54	.65	.10
PHG	.77	.11	−4%	.29	.75	.08	−1%	.11	.74	.10

*Note*: Bold font denotes significant changes compared with HC. Effect sizes and reduction % are calculated in comparison with HC values.

Abbreviations: ACG, anterior cingulate gyrus; AMY, amygdala; BP, binding potential; CFN, ^11^C‐carfentanil; HC, healthy control; HIP, hippocampus; MCG, middle cingulate gyrus; OFC, orbitofrontal cortex; PCG, posterior cingulate gyrus; PHG, parahippocampal gyrus; ROI, region of interest.

### 3.3 Correlation with clinical variables

**FIGURE 3:**
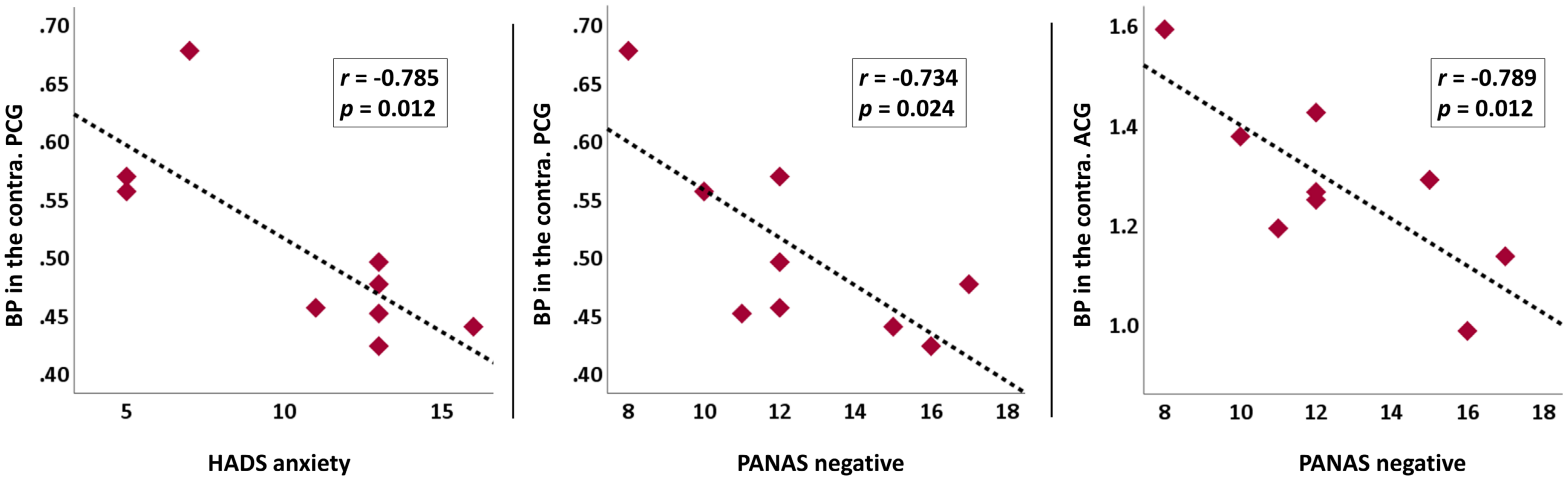
Correlations (uncorrected *p* < .05) of ^11^C‐carfentanil nondisplaceable binding potential (BP) with anxiety and negative affect in the limbic circuit. These correlations did not reach to statistical significance with false discovery rate correction (false discovery rate corrected *p* = .084, corrected for the number of regions of interest). ACG, anterior cingulate gyrus; HADS, Hospital Anxiety and Depression Scale; PANAS, Positive and Negative Affect Scale; PCG, posterior cingulate gyrus.

We found lower CFN BPND in the contralateral posterior cingulate gyrus in patients with TLE and higher anxiety scores (HADS‐A, *r* = −.785, *p* = .012; Figure 3). There was no association with HADS‐D at the uncorrected *p* < .05 level. In patients with TLE, higher negative affect scores measured with the PANAS questionnaire correlated with lower CFN BPND; the higher the negative affect scores, the lower the CFN BPND in the contralateral posterior (*r* = −.734, *p* = .024) and anterior cingulate gyrus (*r* = −.789, *p* = .012; Figure 3). These correlations did not reach statistical significance with false discovery rate (FDR) correction (*p* = .084, corrected for the number of ROIs).

Demographic and clinical variables did not correlate with CFN BPND in any ROIs. The detailed results are shown in Table S1.

## 4 DISCUSSION

We investigated the role of the opioid receptor system in interictal affective disorders in TLE. We observed widely reduced CFN BPND in bilateral frontal lobes and striata in patients with TLE compared to healthy controls. Lower CFN BPND in the posterior cingulate gyrus correlated with more severe anxiety and negative affect within TLE subjects. These results support the involvement of a dysregulated opioid system in the pathogenesis of interictal depression and anxiety.

The opioid system plays a role in both epilepsy and affective disorders. Endogenous opioids are released during spontaneous and provoked seizures and may be involved in seizure termination.^13^, ^25^ Opioids are also released during positive mood induction in healthy individuals.^9^ In people with depression, there is a paradoxical release of opioids during a sustained negative emotion state,^11^ which may contribute to dysregulated emotion processing. As the previous PET studies on seizure‐related opioid release utilized ^11^C‐diprenorphine, which labels multiple subtypes of receptors,^13^, ^25^ it remains unclear whether both seizures and affective disorders involve the same classes of opioid receptors and endorphins. However, given the therapeutic effect of electroconvulsive therapy on depression,^26^ seizure activity may also involve the μ‐opioid neurotransmission system.

The interictal changes observed in our study are novel. We observed that the opioid system is altered interictally and that some of these disturbances correlate with measures of anxiety and negative affect, suggesting that these differences between patients and controls are either induced, by recurrent seizure‐induced opioid release resulting in a downregulation of receptors, or preexisting lower expression of receptors, reflecting a vulnerability to mood disorders. Previous studies analyzing CFN binding in TLE did not include healthy volunteers.^14^, ^15^ Thus, the authors only focused on lateralized changes and could not detect the widespread, bilateral, and largely symmetric reductions of CFN binding in the frontal lobes and striata observed in our study (Figure 1C). Reduced CFN binding can be interpreted as either a reduced density of μ‐opioid receptors or an increased receptor occupancy due to an increased endogenous opioid tone. We have previously demonstrated that endogenous opioids are released during spontaneous seizures but also that the opioid tone returns to baseline in 4–12 h following a seizure. The opioid tone is at normal or low‐normal levels interictally.^13^, ^25^ Our patients were scanned several days (median = 2 days, interquartile range = 19 days) after the last seizure. Thus, an increased interictal endogenous opioid tone is an unlikely explanation for the reduced CFN BPND observed in this study.

Due to these considerations, the reduced CFN BPND in bilateral frontal lobes and striata of patients with TLE may reflect reduced μ‐opioid receptor density. A reduced number of μ‐opioid receptors may be related to receptor desensitization following pulsatile opioid release during chronic repeated seizures. Agonist‐driven desensitization of receptors occurs within minutes after agonist exposure and involves internalization and downregulation of receptors.^27^ Release of endogenous opioids caused a reduced density of opioid receptors in rodent stress experiments^28^ and rodent models of drug addiction.^29^ We speculate that chronic exposure to endogenous opioids released during repeated seizures originating in the temporal lobe could cause a desensitization of receptors in the frontal lobes and striata, areas that are well connected with the epileptic focus.

Given the involvement of the OFC and cingulate gyri in emotional processing and decision‐making,^30^, ^31^ we hypothesized that our findings might relate to psychiatric impairments in TLE. In our study, dysregulation of opioid receptors in the posterior cingulate gyrus correlated with measures of anxiety and negative affect. There were no correlations with other clinical or demographic variables. The posterior cingulate gyrus plays a role in several neuropsychiatric disorders.^32^ An event‐related functional MRI study reported a significant deactivation of the posterior cingulate gyrus in response to threat‐related words in patients with anxiety disorders.^33^ The posterior cingulate gyrus is also involved in chronic pain,^34^ and given the potential similarities between epilepsy and chronic pain, for example, hyperexcitability, our findings in posterior cingulate gyrus might be of relevance. Another study showed decreased functional connectivity in these areas in people with higher levels of anxiety.^35^ The observation of reduced CFN BPND in those with higher levels of anxiety and more negative affect underlines the role of the opioid system in emotion regulation in people with epilepsy.

It is also notable that the correlations with CFN BPND were found only in anxiety or negative affect and not in HADS‐D. There have been arguments on the different characteristics of depressive symptoms in epilepsy from major depression in the general population, and dysphoric mood has been suggested as a potential feature,^36^, ^37^ despite some controversies.^38^ Therefore, our findings would be possibly consistent with such interesting clinical observations on affective symptoms in epilepsy.

It is important to note that other factors in addition to the opioid system play a role in interictal affective disorders, including atrophy of limbic structures, network dysfunction and synaptic disconnection, or alterations to serotonin homeostasis.^6^ The psychosocial effects of seizures, medication, and their impact on the ability to work, drive, and participate in social gatherings additionally contribute to affective disorders.

This study has several limitations. We assessed a small population of patients with TLE. We only observed a correlation with negative affect and levels of anxiety but did not find a correlation with the severity of depression measured with HADS‐D. It should also be noted that only three patients presented suprathreshold depressive symptoms (i.e., HADS‐D ≥ 8), whereas six patients met the threshold for anxiety disorder. Moreover, the correlation results did not survive after FDR correction (*p* = .084), and careful interpretation is necessary. Larger studies might expand on our findings and be able to demonstrate effects related to these symptoms. Additionally, more comprehensive comparisons, for example, TLE without depression versus TLE with depression versus depression without TLE versus healthy controls, should be considered in the future. Like other studies,^39^, ^40^ the group of healthy controls only included males due to the ethical considerations and the potential risk of radiation in females of childbearing age. There is no established evidence on sex differences in CFN BPND, and our results should be carefully interpreted with this limitation. Moreover, like other studies,^39^, ^40^ we did not perform partial volume effect correction. MRI did not show any lesions or focal atrophy except for only one case, so that partial volume effect correction is likely not to alter the findings, although we cannot exclude the possibility of subtle atrophy in the patients with depression. Lastly, we mainly recruited patients with TLE who were undergoing psychiatric evaluation. Some patients also took antidepressants, which might have possibly affected brain CFN BPND. Thus, our results showing reduced CFN BPND in the frontal lobes and striata may not be generalizable to all patients with TLE and may mainly apply to those presenting with psychiatric symptoms.

## 5 CONCLUSIONS

We demonstrated interictal alterations of the opioid system in patients with TLE that were related to higher levels of anxiety and negative affect. Building on these observations and previous research, we propose a hypothetical model of the role of the opioid system in the development of interictal affective disorders. Targeting this system may provide novel treatment avenues for affective disorders in TLE.

## AUTHOR CONTRIBUTIONS

Conceptualization: Jacqueline Foong and Matthias J. Koepp. Formal analysis: Daichi Sone and Marian Galovic. Resources: Marian Galovic, Jim Myers, Georg Leonhardt, and Ilan Rabiner. Writing–original draft preparation: Daichi Sone, Marian Galovic, Matthias J. Koepp, and Jacqueline Foong. Writing–review & editing: Marian Galovic, Georg Leonhardt, Ilan Rabiner, John S. Duncan, Matthias J. Koepp, and Jacqueline Foong. Funding acquisition: Jacqueline Foong. All authors read and approved the final version of the manuscript.

## CONFLICT OF INTEREST

None of the authors has any conflict of interest to disclose.

## Supporting information


**FIGURE S1** ^11^C‐Carfentanil nondisplaceable binding potential (BPND) images of each patient and mean of healthy controls. The number of each patient corresponds to Table 1

**TABLE S1** Correlation analysis of each binding potential with demographical and seizure‐related metrics
