# Supplementary figures and images for: Contribution of the μ‐opioid receptor system to affective disorders in temporal lobe epilepsy: A bidirectional relationship?
Source: Epilepsia. 2022 Dec 28;64(2):420–9. doi: 10.1111/epi.17463 (PMC10107876; doi:10.1111/epi.17463)

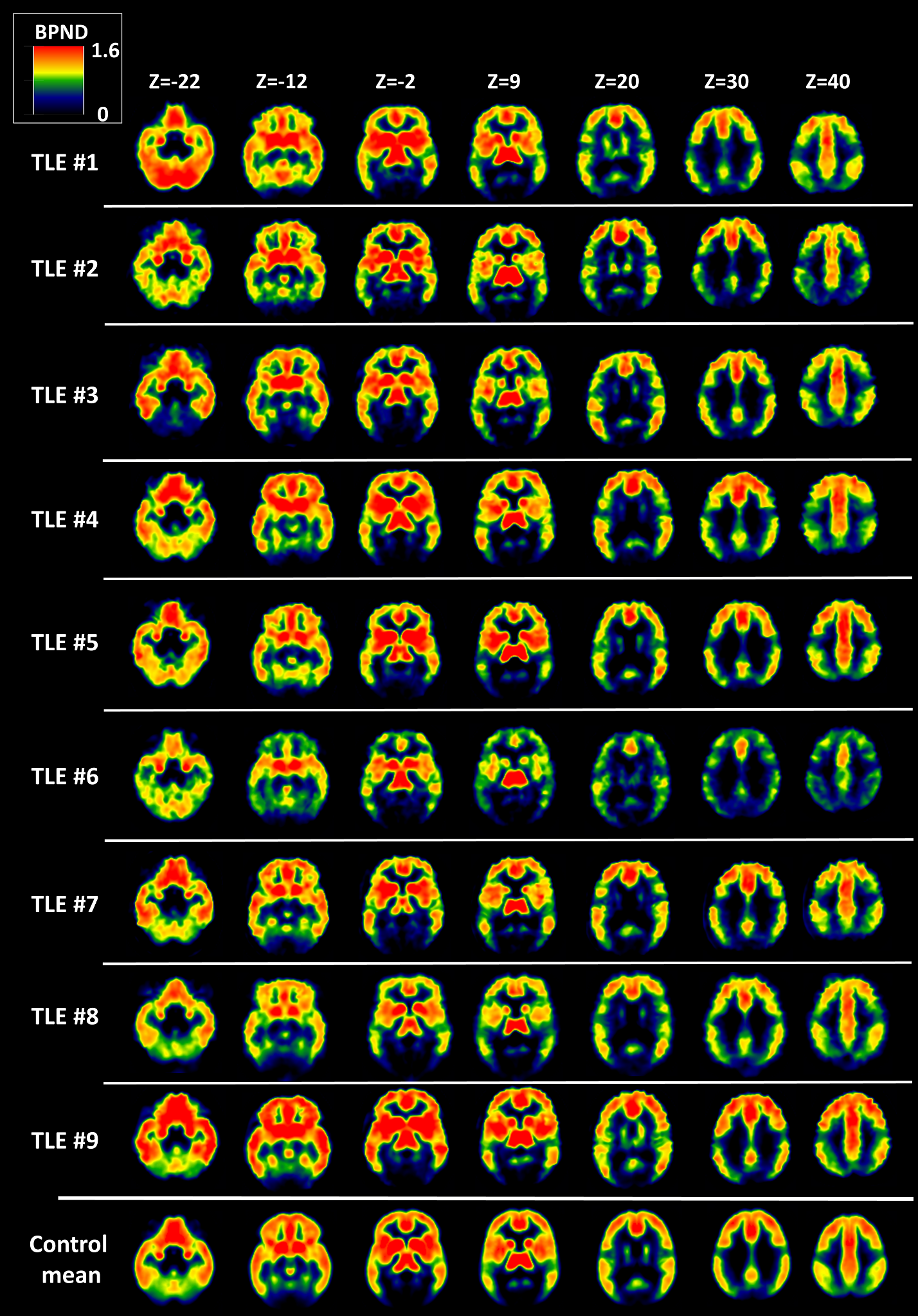

Supplement: Supplementary file 1 — FIGURE S1 11C‐Carfentanil nondisplaceable binding potential (BPND) images of each patient and mean of healthy controls. The number of each patient corresponds to Table 1 [file EPI-64-420-s002.tif]
