# Supplementary material for: Contribution of the μ‐opioid receptor system to affective disorders in temporal lobe epilepsy: A bidirectional relationship?
Source: Epilepsia. 2022 Dec 28;64(2):420–9. doi: 10.1111/epi.17463 (PMC10107876; doi:10.1111/epi.17463)
Supplement: Supplementary file 2 — TABLE S1 Correlation analysis of each binding potential with demographical and seizure‐related metrics [file EPI-64-420-s001.docx]

|  |  | **Ipsilateral** | | | | | | |  | **Contralateral** | | | | | | |
| --- | --- | --- | --- | --- | --- | --- | --- | --- | --- | --- | --- | --- | --- | --- | --- | --- |
|  |  | **OFC** | **ACG** | **MCG** | **PCG** | **AMY** | **HIP** | **PHG** |  | **OFC** | **ACG** | **MCG** | **PCG** | **AMY** | **HIP** | **PHG** |
| Sex (M=0, F=1) | *R* | 0.461 | 0.256 | 0.388 | -0.318 | 0.047 | 0.357 | -0.020 |  | 0.301 | 0.302 | 0.301 | 0.340 | -0.213 | 0.491 | 0.193 |
|  | *P* | 0.211 | 0.506 | 0.302 | 0.405 | 0.905 | 0.346 | 0.959 |  | 0.432 | 0.429 | 0.431 | 0.370 | 0.582 | 0.180 | 0.620 |
| Age | *R* | 0.592 | 0.384 | 0.568 | 0.091 | -0.627 | -0.390 | -0.148 |  | 0.601 | 0.331 | 0.595 | 0.350 | -0.655 | -0.132 | -0.251 |
|  | *P* | 0.093 | 0.308 | 0.110 | 0.817 | 0.071 | 0.299 | 0.705 |  | 0.087 | 0.384 | 0.091 | 0.356 | 0.056 | 0.735 | 0.514 |
| Age at seizure onset | *R* | 0.376 | 0.263 | 0.432 | 0.118 | -0.125 | 0.186 | 0.199 |  | 0.289 | -0.036 | 0.456 | -0.114 | -0.280 | 0.087 | 0.220 |
|  | *P* | 0.318 | 0.494 | 0.246 | 0.762 | 0.749 | 0.632 | 0.608 |  | 0.450 | 0.927 | 0.217 | 0.770 | 0.465 | 0.824 | 0.570 |
| Duration of epilepsy | *R* | 0.059 | 0.012 | -0.009 | -0.050 | -0.321 | -0.432 | -0.292 |  | 0.141 | 0.247 | -0.014 | 0.330 | -0.204 | -0.173 | -0.380 |
|  | *P* | 0.880 | 0.976 | 0.981 | 0.897 | 0.400 | 0.246 | 0.446 |  | 0.718 | 0.521 | 0.971 | 0.386 | 0.599 | 0.656 | 0.313 |
| Presence of FAS (Y=1) | *R* | -0.043 | -0.011 | 0.027 | -0.413 | 0.090 | 0.073 | -0.230 |  | -0.038 | -0.168 | 0.102 | -0.433 | -0.093 | 0.009 | -0.028 |
|  | *P* | 0.912 | 0.978 | 0.946 | 0.269 | 0.819 | 0.851 | 0.552 |  | 0.923 | 0.666 | 0.793 | 0.244 | 0.812 | 0.982 | 0.943 |
| Presence of FBTCS (Y=1) | *R* | 0.260 | 0.201 | -0.211 | 0.119 | 0.424 | 0.529 | 0.440 |  | 0.195 | 0.070 | -0.135 | -0.053 | 0.397 | 0.511 | 0.373 |
|  | *P* | 0.499 | 0.604 | 0.586 | 0.761 | 0.255 | 0.143 | 0.236 |  | 0.615 | 0.857 | 0.729 | 0.893 | 0.290 | 0.160 | 0.322 |
| log10 of interval since last Sz | *R* | -0.484 | -0.344 | -0.364 | -0.276 | 0.555 | 0.467 | -0.021 |  | -0.582 | -0.380 | -0.381 | -0.460 | 0.406 | 0.211 | 0.223 |
|  | *P* | 0.187 | 0.365 | 0.335 | 0.473 | 0.121 | 0.205 | 0.957 |  | 0.100 | 0.313 | 0.311 | 0.213 | 0.279 | 0.585 | 0.564 |
| log10 of overall Sz freq. | *R* | 0.061 | 0.072 | -0.048 | 0.113 | -0.266 | -0.419 | -0.032 |  | 0.243 | 0.144 | -0.004 | 0.099 | -0.058 | -0.315 | -0.230 |
|  | *P* | 0.875 | 0.855 | 0.902 | 0.772 | 0.489 | 0.261 | 0.934 |  | 0.528 | 0.711 | 0.992 | 0.801 | 0.882 | 0.409 | 0.551 |

**Supplementary Table S1.** Correlation analysis of each BP with demographical and seizure-related metrics.
